# High level expression of soluble glycoproteins in the allantoic fluid of embryonated chicken eggs using a Sendai virus minigenome system

**DOI:** 10.1186/1472-6750-7-17

**Published:** 2007-04-05

**Authors:** Teresa Corral, Lorena S Ver, Geneviève Mottet, Olga Cano, Blanca García-Barreno, Lesley J Calder, John J Skehel, Laurent Roux, José A Melero

**Affiliations:** 1 Centro Nacional de Microbiología, Instituto de Salud Carlos III, Majadahonda, 28220 Madrid, Spain; 2 Department of Microbiology and Molecular Medicine, University of Geneva Medical School, CMU, Geneva, Switzerland; 3 National Institute for Medical Research, The Ridgeway, Mill Hill, London NW7 1AA, UK

## Abstract

**Background:**

Embryonated chicken eggs have been used since the mid-20th century to grow a wide range of animal viruses to high titers. However, eggs have found so far only limited use in the production of recombinant proteins. We now describe a system, based on a Sendai virus minigenome, to produce large amounts of heterologous viral glycoproteins in the allantoic cavity of embryonated eggs.

**Results:**

Soluble forms of human respiratory syncytial virus (HRSV) and human metapneumovirus (HMPV) fusion (F) proteins, devoid of their transmembrane and cytoplasmic domains, were produced in allantoic fluids using the Sendai minigenome system. The first step was rescuing in cell cultures Sendai virus minigenomes encoding the proteins of interest, with the help of wild type Sendai virus. The second step was propagating such recombinant defective viruses, together with the helper virus, in the allantoic cavity of chicken embryonated eggs, and passage to optimize protein production. When compared with the production of the same proteins in the culture supernatant of cells infected with vaccinia recombinants, the yield in the allantoic fluid was 5–10 fold higher. Mutant forms of these soluble proteins were easily constructed by site-directed mutagenesis and expressed in eggs using the same approach.

**Conclusion:**

The simplicity and economy of the Sendai minigenome system, together with the high yield achieved in the allantoic fluid of eggs, makes it an attractive method to express soluble glycoproteins aimed for structural studies.

## Background

Over the past decades different expression systems have been developed for production of recombinant proteins. Each of these systems has strengths and weaknesses concerning yield, cost, speed, ease of manipulation and folding and post-translational modifications of the target proteins. *E. coli *is the simplest and most widely used organism for protein expression due to low cost and ease of use but it has serious limitations for expression of mammalian gene products, particularly glycoproteins [1]. Unmodified yeasts, as eukaryotes, are suitable for the production of proteins that do not require mammalian-type glycosylation [2]. However, cultured animal cells still remain the best system in which to produce mammalian glycoproteins, although they have complex nutritional requirements and are sensitive to viral and bacterial contamination [1].

A repertoire of animal viruses has been grown in embryonated chicken eggs since the early 1930's [3]. Eggs have also been used for large-scale production of viruses, aimed at obtaining purified proteins suitable for vaccines or for structural studies [4]. For example, the influenza haemagglutinin [5] (HA) and neuraminidase [6] (NA) ectodomains obtained after protease digestion of egg-grown virus have been crystallized and their structures solved by X-ray diffraction analysis. Chicken eggs have the appealing properties of low cost and ease of manipulation for large-scale production of viruses and recombinant proteins.

Since Sendai virus (SeV, a member of the *Paramyxoviridae *family within the *Mononegavirales *order) replicates very efficiently in eggs, we contemplated the possibility of using this virus as a vector for large-scale production of heterologous glycoproteins in the allantoic fluid of embryonated eggs. Rescue of recombinant SeV [7] and other mononegavirales from cDNA copies of their respective negative single-stranded RNA genomes has been achieved, as well as expression of foreign proteins from the recombinant viruses [8]. However, cDNA cloning and rescue of recombinant paramyxoviruses still entails laborious and time-consuming steps. These difficulties are circumvented in rescuing replication defective minigenomes. These are short negative-stranded RNA molecules in which most of the internal coding sequences of the viral genome have been replaced by a reporter gene (or any other heterologous sequence). Paramyxovirus minigenomes obtained by cDNA cloning can be amplified in transfected cells either expressing a minimal set of complementing viral proteins or superinfected with a wild type homologous helper virus. Although the minigenome can then be amplified in tissue culture, SeV replication is much more efficient in eggs than in cultured cells. Therefore, a SeV minigenome seemed an attractive and versatile vector for the expression of foreign proteins in chicken eggs.

Two heterologous proteins were chosen for proof-of-principle experiments: i) a membrane-anchorless form of the human respiratory syncytial virus (HRSV) fusion (F) glycoprotein [9] and ii) a similar anchorless F protein of the recently identified human metapneumovirus [10] (HMPV). Both, HRSV F and HMPV F are structural proteins that are synthesized as inactive F0 precursors that need to be cleaved proteolytically before becoming membrane-fusion competent. Whereas HRSV F0 is cleaved twice at sites I and II containing furin recognition sequences [11], HMPV F0 is cleaved only once after a single Arg residue [12]. Both proteins are trimers in which each monomer is made of the two chains (F1 and F2) generated after precursor cleavage. A membrane-anchorless HRSV F protein that lacks the C-terminal 50 amino acids (F_TM_-), including the transmembrane and cytoplasmic regions, has been expressed in HEp-2 cells infected with a recombinant vaccinia virus [9] (rVV). HRSV/F_TM_- folds into trimers that are seen under the electron microscope as cone-shaped molecules [13]. An analogous rVV expressing an anchorless form of HMPV/F protein has been made (described in Methods).

## Results and Discussion

**Figure 1:**
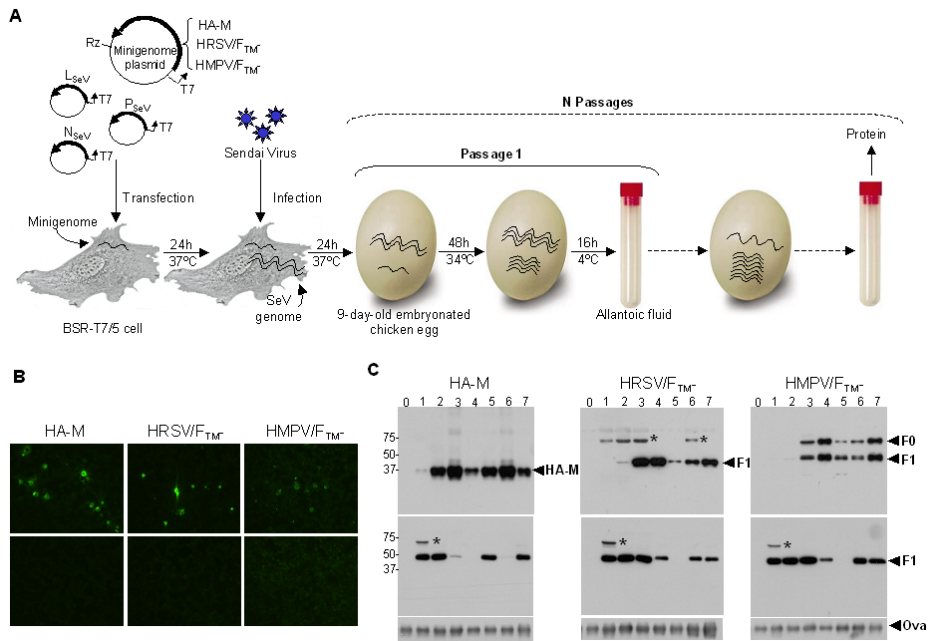
**SeV minigenome expression system**. (A) BSR-T7/5 cells were co-transfected with a minigenome plasmid (SVec/HA-M, SVec/HRSV/F_TM_-, or SVec/HMPV/F_TM_-) and plasmids encoding for SeV L, N, and P proteins, and infected 24 h later with SeV at a m.o.i. of 5. Cells were harvested 24 h.p.i. and 200 μl of culture supernatant were inoculated into 9-day-old embryonated chicken eggs, which were incubated for 48 h at 34°C, and then transferred to 4°C overnight. Allantoic fluid was harvested and subjected to consecutive passages. (B) The immunofluorescence assay was performed in parallel cultures of BSR-T7/5 cells growing in microchamber slides. Cells were transfected with the indicated minigenome along with support plasmids (upper panels) or with the minigenome plasmid only (lower panels) and fixed and stained 48 hours later. (C) Western blot of allantoic fluid from passages 1–7 (lanes 1–7) are compared with allantoic fluid from uninfected eggs (0). Upper panels were developed with antibodies specific for each of the target proteins (HA-M, HRSV/F_TM_-, or HMPV/F_TM_-), middle panels show expression levels of SeV F protein, and lower panels show chicken egg albumin (ova) expression levels, used to standardize the amount of protein loaded in each lane. The asterisk (*) denotes bands that cross-reacted with certain antibodies whose identity has not been investigated. F0 and F1 denote uncleaved F protein precursor and F1 chain, respectively. T7 indicates the bacteriophage T7 promoter and Rz is the hepatitis delta virus ribozyme.

The previously described HA-M-Svec-36-CB119 plasmid [14] (here renamed SVec/HA-M) carries a cDNA SeV minigenome, which encodes an HA tagged SeV matrix protein (HA-M), flanked by a T7 RNA polymerase promoter and the hepatitis delta virus genomic ribozyme (Figure 1A). Transcription of SVec/HA-M by the T7 polymerase results in a positive polarity RNA that, after ribozyme self-cleavage, contains at both ends SeV sequences required for replication and transcription by the SeV polymerase. Therefore, expression of the HA-M protein can be achieved by transfecting BSR-T7/5 cells [15] (which express constitutively the T7 polymerase) with SVec/HA-M and three other plasmids encoding, also under a T7 promoter, the following set of complementing proteins: i) the viral RNA-dependent RNA polymerase encoded by the SeV L gene, ii) the phosphoprotein (P), a cofactor of the viral polymerase and iii) the nucleoprotein (N), a major structural protein that binds tightly to both SeV genome and anti-genome (a replication intermediate of positive polarity), protecting them from RNase degradation.

The HA-M sequence was replaced in the SVec/HA-M plasmid by sequences encoding the F_TM_- forms of either HRSV F or HMPV F proteins (Figure 1A). Cloning was designed to follow the "rule of six" (i.e. that the number of nucleotides in the transcribed minigenome is a multiple of six), a restriction imposed by the number of nucleotides interacting with each N protein monomer in the SeV ribonucleoprotein complex [16]. Then, BRS-T7/5 cells were transfected with SVec plasmids encoding HA-M, HRSV/F_TM_- or HMPV/F_TM_- and the set of support plasmids. Expression of the minigenome-encoded products in transfected cells was confirmed by immunofluorescence of parallel cultures with specific antibodies (Figure 1B). Twenty four hours after transfection, the cells were infected with SeV that provided the required viral products for incorporation of the minigenome ribonucleoproteins into virus particles that would be found, together with wild type SeV particles, in the supernatants of transfected/infected BSR-T7/5 cells, constituting now a mixed virus stock; i.e. virus particles harboring minigenomes or full-length genomes.

The mixed SeV stocks collected 24 hours after SeV infection of BSR-T7/5 cells were inoculated into 9-day-old embryonated chicken eggs for replication within cells of the choriallantoic membrane. Amplification of the SeV minigenome after each passage was evaluated indirectly by Western blot of each recombinant protein. In parallel, multiplication of wild type SeV was evaluated by Western blot of the allantoic fluid with a monoclonal antibody (Mab) specific for the SeV F protein. The amount of allantoic fluid in the Western blots was standardized with an antibody specific for egg albumin (Figure 1C). In general, a major band of the Sendai virus F protein was detected after the first passage in eggs, whereas protein bands of the recombinant proteins were detected only after 2–3 passages. A cycling expression pattern was observed thereafter. Maximal levels of HA-M, HRSV/F_TM_- or HMPV/F_TM_- proteins in allantoic fluids correlated with low expression levels of SeV F protein and vice versa (Figure 1C). This is likely to reflect the competition between minigenomes and full-length SeV genomes for replication and incorporation into virus particles. Minigenomes being shorter are expected to replicate more rapidly than full-length viral genomes. Consequently, minigenomes should quickly outnumber full-length genomes in the infected cells and in the virus particles produced. However, since minigenome amplification and packaging requires helper virus functions, excess minigenome will result in a decrease in minigenome accumulation in the virus stock.

Whereas essentially all the HRSV/F_TM_- was proteolitically processed to the F1 and F2 chains (only F1 was observed with the antibody used in the Western blot) about 50% of the HMPV/F_TM_- precursor remained unprocessed as F0 (Figure 1C). This different behavior is likely to reflect the different proteolytic processing pathways of HRSV and HMPV F0 precursors mentioned above. The SeV F protein was processed efficiently to generate the F1 chain detected with antibody GB5.

**Figure 2:**
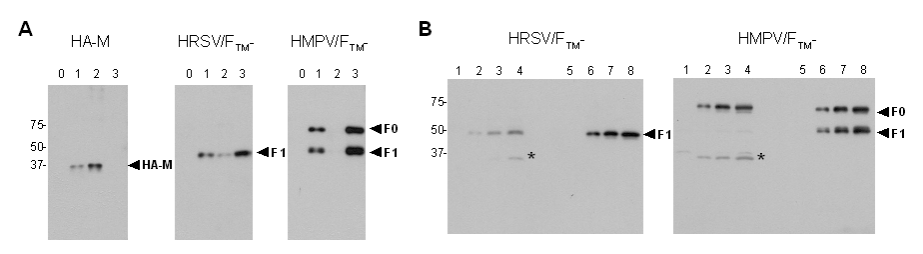
**Analysis of soluble proteins in allantoic fluids**. (A) Allantoic fluids (4.5 ml) from eggs inoculated with mixed stocks of SeV and SeV minigenomes expressing HA-M, HRSV/F_TM_- or HMPV/F_TM_- were centrifuged through a 25% glycerol cushion. Pellets were resuspended in 100 μl of PBS. All fractions were analyzed by Western blot. Five microliters of allantoic fluid from uninfected eggs (0) were compared with 5 μl of allantoic fluid from eggs inoculated with virus stocks encoding the indicated proteins (1), 5 μl of resuspended pellet (2), and 15 μl of the ultracentrifugation supernatant (3). (B) Comparison by Western blot of the expression levels of HRSV/F_TM_-and HMPV/F_TM_- from allantoic fluid or the supernatant of rVV-infected cells. Twenty microliters of culture supernatant from mock-infected HEp-2 cells (1), and 5, 10, or 20 μl of supernatant from rVV-infected cells (2, 3 and 4, respectively) were compared with 20 μl of uninfected allantoic fluid (5) and 5, 10, or 20 μl of allantoic fluid harvested from passage 4 (see Figure 1C) of defective SeV virus-inoculated embryonated eggs (6, 7 and 8, respectively). Asterisk (*) denotes a rVV band that cross-reacts with anti-F specific reagents.

The Western blots of Figure 2A show that the recombinant F_TM_- glycoproteins produced in eggs, in contrast to SeV HA-M, remained essentially unassociated with SeV particles. Thus, after sedimentation of virions from the allantoic fluid, HA-M was found exclusively in the pellet fraction, in agreement with previous demonstration that this molecule can replace the matrix protein of helper SeV in mature particles [17]. In contrast, HRSV/F_TM_- and HMPV/F_TM_- were found mainly, in their respective supernatants. The small amount of HRSV/F_TM_- found in the pellet was probably due to aggregates, generated after complete cleavage of monomers in the protein trimer [11], that may have co-sedimented with SeV particles.

Figure 2B shows comparative Western blots of rVV-infected cell supernatants and allantoic fluids containing either HRSV/F_TM_- or HMPV/F_TM_- glycoproteins. Densitometric quantifications estimated that the concentrations of F_TM_- proteins in the allantoic fluids were 5–10-fold higher than in the cell supernatants. Considering that one egg yields 5–10 ml of allantoic fluid, the amount of F_TM_- protein produced in one egg is equivalent to that found in the supernatant of rVV-infected cells grown as monolayer in a plastic flask of 175 cm^2 ^(~10^8 ^cells). This yield reduces the production cost of F_TM_- proteins about 10 times, considering only the cost of plastic and medium for growing animal cells in comparison with the cost of eggs. However, other significant advantages of embryonated eggs are: i) shorter incubation times, ii) simplicity of manipulations and iii) inexpensive equipment needed for production. These considerations are applicable at the laboratory scale, but other cost calculations may need to be done for industrial processes that require different manipulations and equipment.

**Figure 3:**
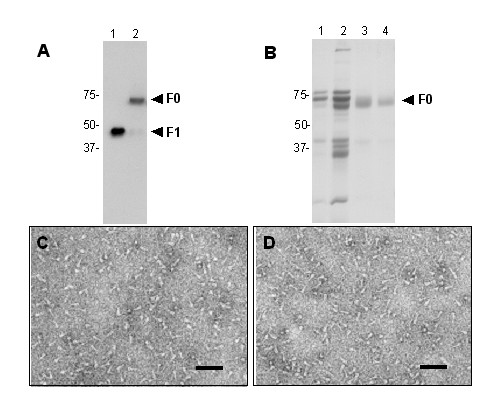
**Purification of HRSV/F_TM_-NN from allantoic fluid**. (A) Allantoic fluid (10 μl) from passage 4 of defective SeV expressing HRSV/F_TM_- (1) compared by Western Blot with passage 4 of HRSV/F_TM_-NN (2). (B)Coomasie-stained SDS-PAGE of allantoic fluid from uninfected eggs (1) compared to allantoic fluid from SVec/HRSV/F_TM_- NN infected eggs (2), HRSV/F_TM_- NN after concentration and affinity chromatography (3), and after a last purification step by gel filtration (4). (C) Electron Microscopy of HRSV/F_TM_- NN purified from eggs or (D) from rVV infected cell supernatant. The bar denotes 50 nm.

As mentioned previously, the small size of plasmids containing cDNA copies of SeV minigenomes facilitates genetic manipulation of the encoded foreign gene. F_TM_- mutants could be easily obtained by site-directed mutagenesis of the minigenomes. As an example, Figure 3A shows the Western blot of a HRSV/F_TM_- mutant (lane 2), compared to F_TM_- wild type (lane 1), in which the basic amino acids of the two furin sites were replaced by Asn residues (NN mutant). Expression of this mutant in eggs generated an anchorless F protein in which all monomers remained uncleaved (F0), as shown previously for an equivalent mutant expressed from rVV [18]. Inhibition of F_TM_- cleavage may be important to maintain this soluble protein unaggregated since, as reported previously, extensive cleavage of its monomers leads to formation of aggregates [11] that sedimented with SeV particles as seen in Figure 2A.

The HRSV/F_TM_- protein produced in eggs has been found indistinguishable in a variety of tests (including reactivity with a panel of MAbs) from that produced in cells infected with rVV. As an example, the NN mutant has been purified to homogeneity (Figure 3B) following the same purification scheme used previously for purification of F_TM_- from the supernatant of rVV-infected cells [11,19]. Electron microscopy of the purified F_TM_- NN protein after negative staining revealed the presence of unaggregated cone-shaped molecules (Figure 3C) similar to those observed in preparations of the same protein from rVV-infected cells (Figure 3D).

Mononegavirales minigenomes expressing foreign reporter genes have been used extensively to dissect *cis*-acting sequences, including promoters, transcription signals and RNA editing signals, as well as *trans*-acting factors required for replication and/or transcription [8]. This report adds to this list the use of mononegaviral (SeV) minigenomes for high yield production of recombinant soluble proteins in chicken eggs. Although only soluble viral glycoproteins have been tested in this paper, there is no reason to believe that the system could not be used for the expression of non-viral glycoproteins, since the expressed molecules were not incorporated into virus particles (as seen in Figure 2A) but were simply secreted to the allantoic fluid.

Coronavirus minigenomes have been used for expression of heterologous proteins in piglets [20] and active gamma-interferon in embryonated chicken eggs [21], although estimation of the amount of foreign protein being expressed was not reported. However, instability of the heterologous gene in the coronavirus minigenome after 4–5 passages in tissue culture cells [20] may be an important limitation for its use in the systematic production of recombinant proteins. In contrast, production of the foreign proteins by the SeV minigenomes reported here was maintained at least after 14 passages in eggs (not shown), although following the cycling pattern depicted in Figure 1C. Nevertheless, high constant production of F_TM_- proteins was achieved by preparing large stocks of passage 3 that were then used to inoculate repetitively eggs for protein purification from passage 4 (see Figure 1C).

Production of active human monoclonal antibodies [22] and single-chain antibodies [23] has been achieved in eggs of chimeric hens. Expression levels were very high, although the recombinant proteins were found in the egg white that has a high ovalbumin concentration. Whereas chimeric chickens may be useful bioreactors for expression of pharmaceutical molecules [24,25], the intense labor- and time-consuming steps involved in obtaining the transgenic chickens limits the system versatility. In contrast, manipulation of the SeV minigenome involves very simple steps that facilitate incorporation of foreign genes and generation of mutant heterologous proteins for comparative studies. Expression of these recombinant proteins in the allantoic fluid, that has a limited number of chicken derived molecules, also facilitates the purification steps of the target proteins.

## Conclusion

The SeV minigenome system described here represents a low-cost, simple and versatile approach for large-scale production of recombinant soluble glycoproteins in the allantoic fluid of chicken eggs.

## Methods

### Cells and Viruses

Hep-2 and CV-1 cells were grown in DMEM medium containing Penicillin and Streptomycin (100 U/ml and 100 μg/ml, respectively) (Cambrex), supplemented with 10% fetal calf serum (DMEM10) (Linus) at 37°C in a 5% CO_2 _atmosphere. BSR-T7/5 cells (a BHK-derived cell line that constitutively expresses the T7 RNA polymerase, a kind gift of K-K. Conzelmann, Munich, Germany) were grown in DMEM10, and G-418 (1 mg/ml) was added every third passage [15] to ensure expression of the T7 polymerase.

Wild type Sendai virus (SeV), strain Z, was propagated in embryonated 9-day-old chicken eggs, as described [26]. Recombinant vaccinia virus (rVV) expressing a soluble form of the HRSV F glycoprotein (F_TM_-), lacking the transmembrane and cytoplasmic domains, has been described [9]. An analogous rVV expressing HMPV/F_TM_- was obtained using the procedure of Blasco and Moss [27]. Briefly, the gene encoding HMPV F was amplified by RT-PCR from total RNA extracted from HMPV infected cells (strain NL/01/99, a kind gift of A.D.M.E. Osterhaus, Rotterdam, The Netherlands) with specific primers and cloned into the pRB21 plasmid [27]. The plasmid pRB21/HMPV/F_TM_- was engineered by substituting the amino acid G489 of the F protein by a stop codon. CV-1 monolayers were infected with the vaccinia virus vRB12 [27] and subsequently transfected with the plasmid pRB21/HMPV/F_TM_-. vRB12 lacks most of the gene encoding vp37, the major protein component of the vaccinia virus envelope, and as a result is unable to form extracellular enveloped virions. Hence, vRB12 is defective in plaque formation under standard conditions. pRB21-derived plasmids carry a complete copy of vp37 and thus, via homologous recombination with vRB12, restore VP37 function. rVV-HMPV/F_TM_- was obtained from the supernatant of infected/transfected CV-1 cells and subjected to three consecutive rounds of plaque purification, and a virus stock was grown in this cell line.

### Plasmids

The plasmid HA-M-SVec-36-CB119 (here SVec/HA-M) contains a SeV-defective RNA genome cloned under the control of the T7 RNA polymerase promoter, and includes the SeV matrix protein gene tagged with the influenza virus haemagglutinin protein epitope [14] (HA-M). Plasmids SVec/HRSV/F_TM_- and SVec/HMPV/F_TM_-, were obtained by replacing the HA-M gene in the vector SVec/HA-M by HRSV/F_TM_- and HMPV/F_TM_-. Briefly, HRSV/F_TM_- and HMPV/F_TM_- were PCR amplified with primers containing XmaI and EcoRI sites, and subcloned in XmaI-EcoRI digested SVec/HA-M. Sequencing of the plasmids confirmed the compliance of the "rule of six" [16]. Plasmids encoding the RNA dependent RNA polymerase (L), the nucleoprotein (N) and the phosphoprotein (P) of SeV under the control of a T7 polymerase promoter (pGEM-4-L, pGEM-4-N and pGEM-4-P/Cstop, respectively) have been described [28,29].

### Rescue of defective viruses

Eight hundred thousand BSR-T7/5 cells were seeded in a 35 mm Petri dish with DMEM10 medium. After 24 h, cells were co-transfected with 7 μg of SVec/HA-M, SVec/HRSV/F_TM_- or SVec/HMPV/F_TM_- plasmids, along with pGEM-L (3.5 μg), pGEM-N (7 μg), and pGEM-P/Cstop (7 μg) using the MBS mammalian transfection kit (Stratagene), following the manufacturer's instructions. Twenty four hours later the cells were infected with SeV in DMEM supplemented with 2% FCS at a m.o.i. of 5, and after 24 h at 37°C the culture medium was harvested, clarified, and kept at -80°C.

In parallel, rescue of the defective minigenome was checked by immunofluorescence of BSR-T7/5 cells growing in microchamber slides. Transfection was done as mentioned in the previous paragraph. Forty eight hours after tranfection the cells were fixed and stained for immunofluorescence with the following primary antibodies: α-HA rabbit polyclonal antibody (Y-11, Santa Cruz Biotechnology), α-HRSV F mouse monoclonal antibody 2F [30] or α-HMPV guinea pig polyclonal antibody (a kind gift of A.D.M.E. Osterhaus, Rotterdam, The Netherlands). Secondary antibodies were fluorescein-linked α-rabbit, α-mouse, or α-guinea pig immunoglobulin antisera (GE Healthcare).

Rescue of the minigenomes in eggs was achieved by inoculating 200 μl of culture supernatant of transfected/infected BSR/T7-5 cells in the allantoic cavity of 9-day-old embryonated eggs. The embryos were incubated at 34°C for 48 h, and then transferred to 4°C. Allantoic fluid was harvested after 16 h in the cold and clarified by low-speed centrifugation. One hundred microliters of the allantoic fluids were used for serial passages in embryonated eggs. Allantoic fluids from the different passages were analyzed by Western blot.

### Western blot

Samples for protein analysis were subjected to 12% SDS-PAGE and electrotransferred to PVDF membranes (Inmobilon-P, Millipore). After blocking with I-Block agent (Tropix), membranes were incubated with one of the following primary antibodies: α-chicken egg albumin monoclonal antibody (C 6534, Sigma), α-HA high affinity rat monoclonal antibody (3F10, Roche), α-Sendai F monoclonal antibody (GB5) or rabbit polyclonal antibodies raised against synthetic peptides HRSV/F_255–275 _or HMPV/F_468–484 _(numbers refer to amino acids included in the peptides). After washing with 0,05%Tween-20 in PBS, the membranes were incubated with horseradish peroxidase-linked α-mouse, α-rat, or α-rabbit immunoglobulin secondary antibodies (GE Healthcare) and developed with ECL.

Quantification of band intensities in Western blots of figure 2B was carried out using the Image J software [31].

### Virus ultracentrifugation

Clarified allantoic fluids were centrifuged through a 25% glycerol cushion in PBS at 35,000 rpm for 3 h at 4°C in a SW40 rotor (Beckman). Pellets were then resuspended in PBS, and analyzed by Western blot.

### Site-directed mutagenesis

SVec/HRSV/F_TM_-NN was obtained by sequential mutagenesis of cleavage site II of HRSV/F_TM_- with the oligonucleotides F387-441+ (5'-CCAATGTAACATTAAGCAACAACAACAACAACAACTTTCTTGGTTTTTTGTTAGG-3') and F441-387- (5'-CCTAACAAAAAACCAAGAAAGTTGTTGTTGTTGTTGTTGCTTAATGTTACATTGG-3') and of cleavage site I with oligonucleotides F309-358+ (5'-GCACACCAGCAGCAAACAATAACGCCAACAACGAACTACCAAGG-3') and F358-309-(5'-CCTTGGTAGTTCGTTGTTGGCGTTATTGTTTGCTGCTGGTGTGC-3'), using the QuickChange Site-Directed Mutagenesis Kit (Stratagene) following the manufacturer's instructions. The plasmid obtained was checked by sequencing.

### Protein purification and electron microscopy

HRSV/F_TM_-NN mutant was purified following procedures used previously for purification of analogous mutants from supernatants of Hep-2 cells infected with rVV [19]. Briefly, allantoic fluid was concentrated and buffer exchanged to PBS by filtration through a 100 kDa pore size membrane (Sartorius) and loaded onto an affinity column made of the monoclonal antibody 2F [30] covalently linked to Sepharose. Protein was eluted at low pH and after neutralization loaded again onto a Superdex 200 10/300 GL (GE Healthcare) column. A homogeneous peak eluted from the gel filtration column was collected and purity checked by SDS-PAGE and Coomassie blue staining. Purified HRSV/F_TM_-NN protein was absorbed onto carbon films and stained with 1% sodium silicotungstate (pH 7.0). A Jeol 1200 electron microscope, operated at 100 kV, was used to view the samples. Micrographs were taken under minimum-dose, accurate defocus conditions to preserve details to ~1.5 nm[32].

## Authors' contributions

TC made the recombinant SVec plasmids carrying the F_TM_- genes and established the expression system. LSV amplified and cloned the HMPV F protein gene. B G-B grew HMPV in tissue culture cells from the original stock and contributed to different cloning steps of HRSV/F and HMPV/F. GM and LR developed the original SeV supporting plasmids and help in setting up the rescue procedure. OC purified the recombinant proteins and LJC made the EM photographs. JJS and JAM were involved in concept development and experimental design.

All authors read and approved the final manuscript
